# Investigation of Risk Factors Associated with Leptospirosis in the North of Iran (2011-2017)

**Published:** 2019-06-22

**Authors:** Ebrahim Sahneh, Ali Delpisheh, Kourosh Sayehmiri, Behnaz Khodabakhshi, Miremad Moafi-Madani

**Affiliations:** ^1^Student Research Committee, Ilam University of Medical Sciences, Ilam, Iran; ^2^Psychosocial Injuries Research Center, Ilam University of Medical Sciences, Ilam, Iran; ^3^Psychosocial Injuries Research Center, Department of Biostatistics, School of Public Health, Ilam University of Medical Sciences, Ilam, Iran; ^4^Department of Infectious Diseases, School of Medicine, Golestan University of Medical Sciences, Golestan, Iran; ^5^Mashhad University of Medical Sciences, Mashhad, Iran

**Keywords:** Leptospirosis, Risk factors, Case-control studies, Iran

## Abstract

**Background:** The risk factors for infection with leptospirosis in Iran have never been studied. We aimed to determine the risk factors of leptospirosis and the epidemiological pattern of this disease in Golestan Province, Iran during 2011-2017.

**Study design:** A case-control study.

**Methods:** This case-control study was performed on the population of patients diagnosed with leptospirosis. Controls were selected from the residents of Golestan province, northern Iran and were matched with the cases for gender, age group, and place of residence. After coding the data collected in checklists, the analysis was performed in SPSS using independent t-test, logistic regression, contingency tables, and Fisher exact test.

**Results:** Eighty-seven cases were diagnosed infected with leptospirosis. Most patients were male (69.0%) and residents of rural areas (82.7%). The three leading risk factors for leptospirosis were exposure to stagnant rice paddy water while having a skin scratch/injury (OR=11.21, 95% CI: 3.02, 43.06), washing the face with stagnant rice paddy water (OR=11.33, 95% CI: 5.12, 25.01), and sighting of rats or rat nest in rice paddies (OR=3.30, 95% CI: 1.01, 11.62).

**Conclusion:** For farmers working in stagnant and muddy waters of rice paddies, occupational protection measures such as wearing waterproof boots, gloves, support, and socks can reduce the chance of infection with leptospirosis. Health education of the people with susceptible occupations about the transmission and prevention methods can also play a key role in controlling this disease.

## Introduction

Leptospirosis is an infectious disease caused by pathogenic bacteria called Leptospira^1^. It is among the most common and most widely spread zoonotic infections in the world. This infection can occur upon direct or indirect exposure to contaminated animals or their urine, especially in areas with alkaline soil and favorable climatic conditions^2-4^. These features may be able to explain the significant increase recently seen in the incidence of this disease in the north of Iran.

Leptospirosis is also known as Weil’s disease, Cane-Cutter fever, Rice-Field fever, Mud fever, Canicola fever, and Harvest fever depending on the location. The primary sources of leptospirosis are cows, horses, dogs, cats, pigs, rats, and other rodents. However, wild animals such as deer, squirrel, fox, weasel, and raccoon, and even marine mammals such as sea lion, and reptiles and amphibians (frogs) may also be infected ^5^.

The most important source of Leptospirosis is the urine of affected animals. *Leptospira interrogans* are able to survive for months in the proximal renal tubule of infected animals without causing any clinical symptoms and spread from there through urine ^6^. Herbivores that have alkaline urine spread more *Leptospira* than those with acidic urine (such as dogs). Infections in humans are usually caused by direct skin contact, particularly through scratches, and also through the mucosa of the mouth while ingesting the contaminated meat, through contact with the mucosa of the eye, nose, and vagina, and respiration of the infected aerosols. Swallowing water while swimming increases the chance of infection. Human to human transmission of leptospirosis is rare ^7, 8^.

Most cases remain mild usually with acute symptoms in the form of headache, muscle ache, fever and chills, nausea, vomiting, and abdominal pain, which lasts for 4 to 7 days. The nonicteric form of the disease is rarely fatal ^7-9^. Major leptospirosis epidemics occurred in Nicaragua in 1995 (with high mortality rates), and in India, Singapore, Thailand, and Kazakhstan in 1997 and 1998 ^6-10^.

If properly diagnosed and treated, leptospirosis has a mortality rate of less than 1%, with higher rates for more advanced cases. The prognosis of the disease depends on the virulence and the general condition of the patient but is generally favorable. The elderly, pregnant women and people with severe jaundice or renal dysfunction have higher mortality rates^11^.

Leptospirosis can be diagnosed by testing blood or serum levels for *Leptospira* -specific antibody at least five days after the onset of clinical symptoms. The gold standard for diagnosis of leptospirosis is the Microscopic Agglutination Test (MAT). ELISA test of serum IgG and IgM levels can also provide highly accurate diagnosis^12^. The DNA test based methods can also be useful in the diagnosis of leptospirosis^13^.

In most parts of the world, the prevalence of leptospirosis is very low, but many cases of the disease remain undiagnosed and unreported. Farmers, sewage workers, veterinarians, slaughterhouse workers, fishermen, livestock farmers, and miners are the groups most exposed to this disease. People who sail and swim in infected waters are at also at the risk of getting Leptospirosis ^14^.

Risk factors for leptospirosis vary with the country. In France, for example, canoeing is the most important risk factor^15^. In Germany, people who work in the forestry industry or have pet mice are most likely to get Leptospirosis^16^. In Thailand, however, farming jobs that involve walking and working in paddy fields (barefooted) are the main factor of the disease^17^.

Leptospirosis is endemic in the north of Iran, in the provinces of Gilan, Mazandaran, and Golestan ^18^. The health system personnel have little information about this disease. Leptospirosis may become a serious threat against public health in the future if it is left unattended. According to the survey, risk factors of leptospirosis infection have not yet been measured in Iran. Given the importance of Leptospirosis, the findings of this study can contribute to the promotion of public health in this region.

## Methods

This research was designed in the form of an analytical population-based case-control study. The cases were the patients diagnosed with leptospirosis in Golestan Province (Iran) from the beginning of Apr 2011 to the end of Mar 2017. The diagnosis confirmation criteria were the culture samples or PCR testing positive or IFA titer is equal to or greater than 1.80.

For each case, one person from the population of Golestan Province was selected as control. Golestan Province with an area of 22033 km^2^ is located on eastern north of Iran. It is characterized by various climates and climate diversification. As a whole, this province climate is categorized as Mediterranean mild and humid climate.

The exclusion criterion for the controls was infection with leptospirosis in the past or at the time of the study. All controls were matched with the cases for gender, age group (0-10, 20-10, 20-30, 30-40, 40-50, 50-60, 60-70, and >70), and place of residence (required to be within 500-m radius). The total number of leptospirosis cases recorded at the health centers of Golestan Province during the 2011-2017 period was 142. Of these, 89 cases confirmed diagnosis (by PCR, IFA, or cultures testing positive) were selected for the study. Two patients were excluded because of inaccessibility.

After preparing the list of patients and obtaining their home addresses, they were interviewed in person and the checklist of risk factors was completed. The checklist is organized in 4 pages, in which 72 variables included regarding the risk factors of leptospirosis infection. The questions include variables such as people residential information, their jobs, the history of being contacted to stagnant water, the history of any skin injuries, any contact with animals, etc. While visiting the cases, the demographic data of the neighbors were also collected and examined to choose the control subjects. The checklist of risk factors for controls was also completed. From each control, 4 cc of blood sample was collected and immediately transferred to the laboratory while maintaining the cold chain. In the laboratory, at least 1.5 ml of the serum was isolated and frozen at -35 °C for later tests. All cases and controls were asked to provide written consent.

After collecting samples from 11 cities of Golestan province and transferring them to the reference laboratory at Gonbad-e Kavous Health Center, agglutination test was performed using the Rapid Test Kit manufactured by Pars Faravard Co. (approved by the Reference Health Laboratory of the Ministry of Health of Iran). Among the controls, 4 tested positive for leptospirosis and excluded. The excluded controls were replaced with negative controls with similar characteristics. After coding the checklist data, the collected information was entered into the SPSS (ver.16, Chicago, IL, USA) for analysis. Data analysis was performed using logistic regression, independent t-test, contingency tables, Fisher exact test, and odds ratio calculation.

The mathematical formulation of this regression model was estimated as follows.

$$ln\frac{\text{p}}{1-\text{p}}=-1\;+\;2\text{X}1\;+\;2\text{X}2\;+\;2\text{X}3\;=\text{ f}\left(\text{x}\right)$$

Risk probability $\frac{e^{\text{f}\left(\text{x}\right)}}{1+e^{\text{f}\left(\text{x}\right)}}=\;\frac{e^{-1\;+\;2\text{X}1\;+\;2\text{X}2\;+\;2\text{X}3\;}}{1+e^{-1\;+\;2\text{X}1\;+\;2\text{X}2\;+\;2\text{X}3\;}}$ Probability of infection P=P_r_ (y=1)

## Results

The total number of patients was 87 and they were distributed over 11 counties of Golestan province. Controls were matched with the cases for age group, gender (male, female) and place of residence (neighborhood).

Among patients, the demographic classes with the highest frequencies were male (69%), residence in rural areas (82.7%), age groups of 31-40 and 41-50 (25.2% each), and Persian ethnicity (55.2%). The age group with the least frequency was 10-20 yr (2.3%). The counties of Gorgan (36.7%) and Galikesh (1.2%) had the highest and lowest frequency of patients, respectively. The level of education seems to be related to the incidence of leptospirosis, as grade school education was most frequent (37.9%) and bachelor’s degree was least frequent (3.4%) among patients.

Geographic location (place of residence) of the subjects could not be analyzed as a risk factor, because each case was matched with one control living in the vicinity. From the 72 risk factors and independent variables studied, logistic regression test (Table 1) found rice farming occupation (OR=12.89, 95% CI: 5.46, 30.44), working in wet environment (OR=7.65, 95% CI: 3.58, 16.35), daily sighting of rats, rat droppings, and rat nests in the residence (OR=3.61, CI=1.14, 11.35), washing the face with stagnant rice paddy water (OR=11.83, 95% CI: 3.74, 37.43) , and exposure to stagnant rice paddy water while having a skin scratch/injury (OR=18.00, 95% CI: 5.00, 60.00) as the factors with the highest risk. No interaction was found between farming occupation, rat sighting, washing the face with rice paddy water, and exposure to rice paddy water while having an injury.

**Table 1:** Risk factors for infection with leptospirosis in Golestan province (2011-2017)

**Variables**	**Cases**	**Controls**	**OR (95% CI)**	***P*** **value**	**Adjusted OR** **(95% CI)**	***P*** **value**
Occupation						
Farmer (rice)	69	22	11.36 (5.57, 23.01)	0.001	12.89 (5.46, 30.44)	0.001
Farmer (other crops)	37	23	2.05 (1.08, 3.89)	0.038	1.48 (0.67, 3.29)	0.329
Work environment						
Wet working environment	71	31	8.01 (3.99, 16.10)	0.001	7.65 (3.58, 16.35)	0.001
Sighting of rats (at least once a week)	18	5	4.27 (1.51, 12.11)	0.006	0.98 (0.45, 2.12)	0.963
Presence of livestock at work	31	19	1.98 (1.01, 3.87)	0.065	1.41(0.65, 3.06)	0.375
Residence environment						
Sighting of rats (at least once a day)	15	5	3.41 (1.18, 9.86)	0.030	3.61 (1.14, 11.35)	0.028
Exposure to natural water						
Swimming	23	12	2.24 (1.03, 4.86)	0.058	1.92 (0.58, 6.38)	0.284
Washing the face	69	18	14.69 (7.05, 30.60)	0.001	11.83 (3.74, 37.43)	0.001
skin scratch/injury	34	3	17.96 (5.25, 61.42)	0.001	18.00 (5.00, 63.00)	0.001
Exposure to animals						
contact with dogs	36	21	2.21 (1.15, 4.25)	0.023	1.18 (0.61, 2.28)	0.606
Contact with livestock	16	4	4.67 (1.49, 14.62)	0.008	1.41 (0.65, 3.06)	0.375

The highest risk ratio was related to exposure to stagnant rice paddy water while having a skin scratch/injury. Washing the face with other sources of water such as spring water, running river water, and pool water were not recognized as a risk factor for leptospirosis. Moreover, no significant relationship was found between leptospirosis and the source of drinking water and dietary habits.

Of the four independent variables examined as preventive factors (Table 2), only wearing boots when exposed to stagnant rice paddy water (OR=0.07, 95% CI: 0.02, 0.22) had such effect. In fact, not wearing boots when exposed to this water corresponds to 13 times increase in the chance of infection with leptospirosis.

**Table 2:** Risk factors and preventive factors for infection with leptospirosis in Golestan province (2011-2017)

**Risk factor**	**Cases**	**Controls**	**OR (95% CI)**	***P*** **value**	**Adjusted OR** **(95% CI)**	***P*** **value**
Wearing normal shoes	43	71	0.22 (0.11, 0.43)	0.001	0.92 (0.52, 1.61)	0.772
Wearing sandals/slippers	31	64	0.19 (0.10, 0.38)	0.001	0.89 (0.48, 1.35)	0.622
Wearing boots	16	66	0.07 (0.03, 0.14)	0.001	0.07 (0.02, 0.20)	0.001
Wearing gloves	18	52	0.17 (0.09, 0.34)	0.001	1.15 (0.37, 3.60)	0.804

After using the backward conditional method to examine the statistically significant variables to find the best model, exposure to stagnant rice paddy water while having a skin scratch/injury (OR=11.21, 95% CI: 3.02, 43.06), washing the face with stagnant rice paddy water (OR=11.33, 95% CI: 5.12, 25.01), and sighting of rats or signs of rats (OR=3.30, 95% CI: 1.01, 11.62) are the optimized risk factors for infection with leptospirosis (Table 3).

**Table 3:** Comparison of the optimized risk factors for leptospirosis in Golestan province (2011-2017) using the logistic regression model

**X**	**Variables**	**Regression** **coefficient (β)**	**OR (95% CI)**	***P*** **-value**
X_3_	Sighting of rats, rat droppings, and rat nests (at least once a week) in work environment	1.2	3.30 (1.01, 11.62)	0.001
X_2_	Washing the face with rice paddy water	2.4	11.33 (5.12, 25.01)	0.001
X_1_	Exposure to stagnant rice paddy water while having a skin scratch/injury	2.4	11.21(3.02, 43.06)	0.001

## Discussion

This study is the first to investigate the risk factors for human leptospirosis in Iran. In this study, the prevalence of leptospirosis in Golestan Province, Iran (for the 2011-2017 period) was estimated at less than 1 per 100,000 population (0.76).

Each year, nearly one million people get severe leptospirosis and 60,000 people die from this infection. In 1998, the prevalence of this disease in France was 0.44 per 100,000 population^19^.

Risk factors for leptospirosis vary with the country. A nation-wide study conducted in France in 2000 found that canoeing (OR=15.5) and exposure of skin scratch/injury to stagnant water (OR=7) were the leading risk factors for this disease^15^. The results of the present study also suggested that having skin injuries in the lower limbs can be considered a major risk factor for getting this infection from contaminated waters.

In Germany, contact with pet rats (RR=13.89) and forestry occupation (RR=9.2) were reported as the most important risk factors for leptospirosis^16^. In Iran, leptospirosis is enzootic in the rodent population of the Northern provinces^20^. Given the high rates of infection in rodents, considered the primary source of this disease, the risk of transfer to humans and other animals cannot be ignored ^21^. The results of this study indicated that the presence of rats, their droppings, or their nests in rice paddies increase the risk of infection with leptospirosis.

In Argentina, a study for the period 1999-2005, people with rural occupations (OR=3.4), exposure to surface water (OR=2.1), and exposure to flood water (OR=4.4) had the highest probability of infection with leptospirosis ^22^. The results of the present study also showed that the disease is most frequent in rural areas (82.7%) and among rice farmers (75.8%) (OR=12.8).

In northern parts of Thailand, working barefoot in rice fields and paddies had the highest odds ratio (OR = 7.1) for leptospirosis ^17^. Thailand and northern parts of Iran have similar risk factors for leptospirosis. Perhaps, this similarity is due to the similarity of ecological and climatic conditions, but more likely, it can be attributed to the presence of vast rice fields, farmers being accustomed to risky work habits (working barefooted in rice paddies), and also the presence of large sources of the disease.

Contact with livestock and animals such as dogs increase the risk of leptospirosis by 4 and 2 times, respectively. This is notable because previous studies and seroepidemiologic investigations in Golestan have reported numerous cases of infection with leptospirosis in livestock of this area ^23^.

Leptospirosis is difficult to control from a public health perspective. In some European countries, especially France, people in certain areas and occupations receive inactivated vaccine ^15^. Considering the variety of serogroups, this vaccine could be less effective than expected, but more research in this area is still needed.

Given the retrospective nature of this study, to reduce information bias, the researchers tried to rely on more precise definitions of exposure with more emphasis on being tangible and objective. Moreover, the information contained in the health records of the central health office of the province was used for this purpose.

To minimize the impact of interviewer bias, the researcher’s defined precise standardized methods for data collection, provided guidelines and training for interviewers, and blinded them to research objectives.

## Conclusion

Using protective equipment such as waterproof gloves, rubber boots, and support or socks when exposed to stagnant and muddy waters (especially rice fields and paddies) can prevent the infection with Leptospirosis. Designing new clothes specifically for working in these fields can also be helpful in this regard. Providing health education about transfer and prevention methods for the susceptible population, that is, the people highly exposed because of their lifestyle or occupation can also play a key role in controlling this disease.

The control of rodent populations and supplementary measures such as vaccination of susceptible human population, livestock and other perissodactyla animals can be recommended as second-level strategies for controlling this infection in the areas where it could be a public health problem.

## Acknowledgements

We would like to express our sincere gratitude to the staff of health centers of Golestan Province and all people who contributed to this research.

## Conflict of interest

The authors declare that there is no conflict of interests.

## Funding

This study has been supported financially by the Vice Chancellor for Research and Technology of Ilam University of Medical Sciences. The research code of ethics is IR.MEDILAM.REC.1397.115).

## Highlights

- This study is the first to investigate the risk factors for human leptospirosis in Iran.
- The highest risk ratio was related to exposure to stagnant rice paddy water while having a skin scratch/injury.
- Not wearing boots when exposed to this water corresponds to 13 times increase in the chance of infection with leptospirosis.
